# High-Performance Capillary Electrophoresis for Determining HIV-1 Tat Protein in Neurons

**DOI:** 10.1371/journal.pone.0016148

**Published:** 2011-01-07

**Authors:** Satish L. Deshmane, Ruma Mukerjee, Shongshan Fan, Bassel E. Sawaya

**Affiliations:** 1 Department of Neuroscience and Center for Neurovirology, Temple University School of Medicine, Philadelphia, Pennsylvania, United States of America; 2 Laboratory of Molecular Studies of Neurodegenerative Diseases, Department of Neurology, Temple University School of Medicine, Philadelphia, Pennsylvania, United States of America; 3 Department of Pathology and Laboratory Medicine, Temple University School of Medicine, Philadelphia, Pennsylvania, United States of America; George Mason University, United States of America

## Abstract

The HIV-1 protein, Tat has been implicated in AIDS pathogenesis however, the amount of circulating Tat is believed to be very low and its quantification has been difficult. We performed the quantification of Tat released from infected cells and taken up by neurons using high performance capillary electrophoresis. This is the first report to successfully measure the amount of Tat in neurons and places Tat as a key player involved in HIV-associated neurocognitive disorders.

## Introduction

HIV infection is pandemic with more than 30 million people infected worldwide. In the USA following the onset of AIDS, 10–15% patients per year develop HAND, a neurocognitive and motor abnormality during the later stage of infection [1]. However, in recent years, *in vitro* and *ex vivo* studies have attempted to characterize the mechanisms that underlie the relationship between HIV infection and HAND. Available data suggest that the mechanism(s) leading to damage in the brain of AIDS patients might involve the combined effects of more than one neurotoxic factor [2]. In particular, evidence suggests that viral proteins (e.g., Tat) secreted from HIV-1 infected cells [3] are among these factors. Although the use of HAART reduces the frequency of HAND, this treatment might be less efficient in brain tissues and therefore in the treatment of HAND [4]. In fact, the quality of life of some HIV patients continues to be diminished by residual, milder forms of neurocognitive impairment. Tat is a viral transactivator of the HIV-1 promoter [5], [6]. It binds the cyclin T1 component of the positive transcription elongation factor b, recruits cyclin-dependent kinase 9 to elongating HIV transcripts and induces phosphorylation of the C-terminal domain of RNA polymerase II by Cdk9 [7]. Tat is mainly active in the nucleus and is secreted at high-levels *in vitro* [8]. Secreted Tat can cause direct or indirect injury to neurons, thus Tat may contribute to neurological impairments observed in HIV patients on successful HAART regimens. The neurotoxicity of Tat involves prolonged elevations in intracellular calcium [9] followed by an increase in reactive oxygen species and activation of the apoptotic pathway [10]. Tat also promotes activation of monocytes, macrophages and astrocytes triggering the release of inflammatory factors, which can lead to neuronal damage [11]. All attempts leading to inhibit Tat-mediated neurodegeneration both *in vitro* and *in vivo* have failed. It has been shown that at a concentration as low as 1 nanomolar and 2 to 20 femtomolar, HIV-1 Tat can significantly induce apoptosis of PC12 or rat neuronal degeneration, respectively [12]. In addition, Tat induces apoptosis in human neuroblastoma cells [13], in human fetal neurons [14] and in embryonic rat hippocampal neurons [12]. However, the exact amounts of Tat released from HIV-infected cells or taken up by non-infected cells remain unclear. With the introduction of proteomics and the development of these techniques mainly high performance capillary electrophoresis (HPCE) this measurement is now possible.

In this study, we used HPCE to determine the amount of Tat taken up by neuronal cells that can lead to neuronal degeneration. This information may be valuable for the development of therapeutic agents that protect the CNS neurons from toxic viral factors thus lessening the severity of HAND.

## Methods

### High performance capillary electrophoresis (HPCE)

HPCE allows for separation of molecules based on their sizes, structure, charges and hydrophobic potential. For fluorescence derivatization, 10 µl of recombinant Tat protein or biological samples, 10 µl of phosphate buffer and 0.5 µl of 60 mM 4-Fluoro-7-nitro-2, 1,3-benzoxadiazole (NBD-F) were used. The mixture was heated at 55°C for 15 min in the dark. The Beckman P/ACE MDQ capillary electrophoresis instrument (Fullerton, CA) equipped with a laser-induced fluorescence detector was used for quantitative analysis of Tat protein in biological samples. LIF detection was performed in an uncoated fused silica CE column of 50 µm inner diameter and 60 cm in length with 50 cm from inlet to the detection window (Polymicro Technologies, Phoenix, AZ). The injection was applied hydro dynamically at a pressure of 0.4 p.s.i. for 8 seconds. The separation voltage was 25 kV. Data were collected and processed using the Beckman P/ACE 32 Karat software version 7.0.

### Cell culture and transfection assays

Human microglia and neuroblastoma (SH-SY5Y) cell lines [15], [16] and primary human neurons (HN) [purchased from ScienCell Research Laboratories (Carlsbad, CA)] were maintained in DMEM +10% FBS. Confluent SH-SY5Y cells were re-plated at 1–5×10^5^ cells/ml and induced to differentiate by treatment with 10 µM retinoic acid (Sigma, St. Louis, MO) for 7 d with medium changes every two days. For all of the experiments, cells were serum starved for 6 h in the presence of 10 mM RA prior to treatment with rTat or transfection after which complete fresh media was added.

Cells were transfected with 0.1 µg of LTR-luc reporter plasmid or treated with increasing amount of recombinant Tat protein (rTat), co-transfected with 0.25 µg of Tat expression cDNA or transduced with Ad-null or Ad-Tat [10]. The cells were also transfected with 1.0 µg of CMV-β-galactosidase plasmid using Nucleofector kit V (Amaxa). The amount of DNA used was normalized with pcDNA_3_ vector plasmid. Cell extracts were prepared 48 h after transfection and luciferase and β-gal determined. All values were normalized against β-gal values measured as previously described [16].

### HIV-1 infection

The human U-937 monocytic cell line [17] was maintained in RPMI +10% FBS, 100 units/ml penicillin, 50 µg/ml streptomycin-G. Cells in log phase were infected with JR-FL strains of HIV-1 as follows [18]. Fifty nanograms of p24-containing virus stock were added to every 1×10^6^ cells. Cells were incubated with virus stock in a small volume of serum free media for 2 h at 37°C. The cells were then washed twice with PBS and fresh medium containing 2% of FBS was added (500,000 cells/ml).

P24 ELISAs were performed for tissue culture supernatants as described by the manufacturer (Coulter-Immunotech, Wesbrook, ME). Each sample was assayed over a 10,000-fold range of dilution, to ensure quantitation, was based on an OD value within the linear range of the standards. Neuronal cells were treated (infected) with supernatant prepared from HIV-1-infected cells (concentration  = 10 MOI) for 24 hr and processed as described in the Results section.

### Cell Extracts Preparation

SH-SY5Y cells were cultured under different conditions (± rTat, or ± supernatant prepared from HIV-infected or control cells) after which cells were lysed in lysis buffer [10]. Protein lysates were diluted to the appropriate/required volume and used for HPCE assay.

### Cell death assays

SH-SY5Y cells were mock-treated or treated with 1 pg/ml of rTat for 24 hrs. The cells were then collected and incubated with 0.4% trypan blue dye (Invitrogen Life Technologies) for 4 min, before washing with PBS and counting. Unstained (viable) and stained (dead) cells were counted separately using hemacytometer.

In parallel, untreated or Tat-treated SH-SY5Y cells were also plated, fixed and stained with mouse anti-microtubule-associated protein-2 (MAP-2) or anti-Tubulin mAb (Cell Signal) followed by Cy3-conjugated rabbit-anti-mouse secondary Ab (Abcam). Scion Image software was used to quantify MAP-2 reactivity after Tat treatment.

### Neurites Outgrowth Analysi

SH-SY5Y cells (mock-treated or treated with 1 pg/ml of rTat) were grown on chamber slides for 24 hrs, fixed in 4% paraformaldehyde, washed and then stained for 3 min in a 4% Trypan blue methanol/acetic acid/water solution. Anti-tubulin antibody was also used. The cells were observed at different magnifications through phase contrast microscopy (convolution). The images acquired on different samples were analyzed to quantify 3 different parameters: sample area covered by cells, neurite extension to cell area ratio, and mean of single neurite length. Single and total neurite analyses were done on areas of 0.30 mm^2^ in order to obtain a more accurate measure of neurite length. The experiment was performed in triplicate.

Statistical analyses were assessed by 1-way ANOVA within groups (same time of cell culture, same culture medium), followed by a Tukey posttest using GraphPad Prism version 4.00 for Windows, GraphPad Software (San Diego CA, USA) to evaluate neurite lengths. Two-way ANOVA was used for comparisons between groups, followed by a Bonferroni post-test. Differences were considered statistically significant for *p* < 0.05.

### 2DE-based Differential Proteomics Technology

U-937 cells were infected with HIV-1 and the supernatant was added to neuronal cells. Proteins from the Tat-detected peak were collected and separated by 2DE gel via two IPG strips (pH of Tat is 6.12) followed by Sypro-Ruby fluorescence staining. Next, In-Gel Trypsin Digestion-Differentially expressed spots were excised and destaining of the excised gel pieces was performed. The detected spots were analyzed by MALDI-TOF. Matching the calibrated peptide mass values within NCBInr protein databases identified Tat protein.

## Results and Discussion

Several reports demonstrated the presence of HIV-1 Tat proteins in human neurons *in vitro* and *in vivo* [18] however, the exact amount of Tat released from HIV-infected cells and subject to neuronal uptake remained unclear. We have now addressed this question using high performance capillary electrophoresis (HPCE). First, we measured the amount of Tat released from the human monocytic cell line, U937, infected with JR-FL strain of HIV-1 and taken up by the neuronal cell line, SH-SY5Y. Briefly, HIV-1 was added to 1×10^6^ U937 cells for 10 days [19]. On day 10 post-infection, supernatant from the U937 cells was collected and ultracentrifuged to remove virus, while leaving Tat and other secreted viral proteins. Three ml (10 MOI) of this supernatant was added to SH-SY5Y cells. After 24 h, supernatants and cell lysates collected from HIV-1-infected U937 or non-infected SH-SY5Y, respectively, were processed for detection of Tat by HPCE [20].

**Figure 1:**
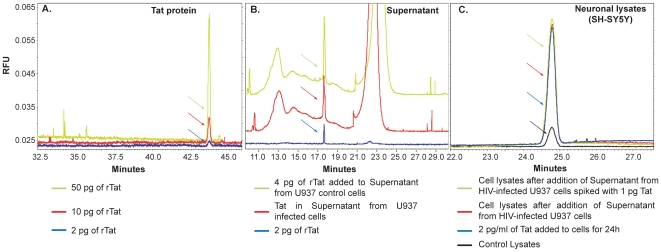
High performance capillary electrophoresis analysis of Tat protein. ***A***. Electropherograms showing different concentrations of rTat in order to examine the sensitivity of the assay. ***B***, ***C***. Representative electropherograms comparing the amount of Tat in supernatant from infected cells or from lysates from uninfected neuronal SH-SY5Y cells to which the supernatant was added.

To first establish a standard curve for calibration, solutions containing recombinant Tat protein (101aa) (rTat) (*highly pure toxin-free Tat purchased from Immuno Diagnostics*, *Inc.*; *Woburn*, *MA*), were prepared at concentrations ranging from 2 to 50 pg and concentrate in 10 µl. As shown in Fig. 1A (blue), approximately 2 pg represent the assay's detection limit for Tat. Next, the amount of Tat was measured in the supernatant from infected U937 cells (Fig. 1B, red) and was found to be approximately 2 fold greater than the rTat at 2 pg in the standard curve (blue). To determine the approximate concentration of Tat in U937 cells, 4 pg of rTat was added to the U937 infected supernatant (green). Tat was also detected in neuronal extracts from neurons exposed to supernatant from HIV infected U937 cells (Fig. 1B, panel C, red). To calculate the amount of Tat in lysates, non-infected extracts were spiked with 1 pg of rTat (Fig. 1C, green). The amount of Tat detected in SH-SY5Y neuronal cells was about 2 pg/ml (Fig. 1C, red) as compared to spiked lysates (Fig. 1C, green). As a negative control, proteins from non-infected SH-SY5Y cells were used (Fig. 1C, black). Uptake of Tat was also measured using SH-SY5Y cells exposed to 2 pg of rTat for 24 h (Fig. 1C, blue) after which cell proteins were prepared. Tat released from infected cells should be equal to 3 to 4 pg based on the conclusion that at least 50% of the released Tat is taken up by neuronal cells [8]. This conclusion correlates with the results obtained and shown in panel B (red). Note that the rationale for choosing SH-SY5Y cells is that they mirror pathways involved in neurodegenerative process associated with HIVE [21]. It should be added that difference in time observed in Fig. 1 is due to the nature of samples analyzed, where in sample 1, rTat was the only component to be analyzed while in other panels Tat was issued from either the supernatant of infected cells or from cell extracts after adding the infected/isolated supernatant. Thus, the presence of other proteins delayed the release of Tat and the presence of additional peaks (e.g. Fig. 1B). Note that analyses of the additional peaks from Fig. 1B did not reveal the presence of Tat protein and therefore these peaks do not represent dimers or multimers Tat.

**Figure 2:**
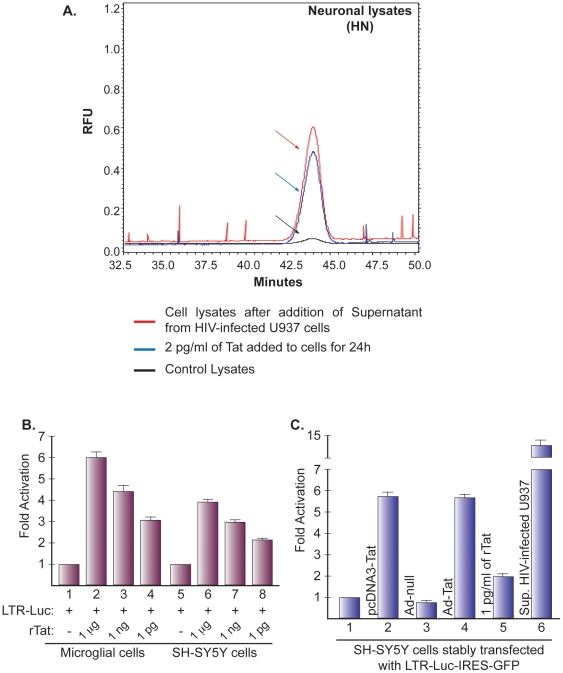
Measurement of Tat in primary human neurons (HN) and ability of rTat to activate HIV-1 gene expression. ***A***. Representative electropherograms comparing the amount of Tat in supernatant from infected cells (red) or from extracts from uninfected (black) HN cells to which the supernatant was added. As a control, extracts were also prepared from rTat-treated cells (blue). ***B***. Human microglia and SH-SY5Y cells were transfected with 0.5 µg of LTR-Luc alone and treated with different concentrations of rTat. ***C***. SH-SY5Y cells expressing the LTR-luc-IRES-GFP were transfected with Tat expression plasmid, infected with Ad-null or Ad-Tat, or treated with rTat (1 pg/ml) or supernatant from infected cells. Cell extracts were prepared 24 h (microglia) or 48 h (SH-SY5Y) after transfection and luciferase and β-gal determined. All values were normalized against β-gal and represent mean of at least 3 experiments.

Similar results were obtained when using primary human culture of neurons. Briefly, cells untreated (control), rTat-treated (2 pg/ml) or treated with supernatant prepared from HIV-infected cells were processed and the amount of Tat in these cells was measured. Data shown in Figure 2 confirmed results presented in Figure 1 and show that Tat was detected in treated cells and not in the control. Similar results were obtained when using the CSF from an HIV-1 negative, and an HIV-1 positive patient (data not shown).

In order to confirm the presence of Tat in the cells, primary human neurons were treated with supernatant from HIV-infected cells, using 2DE-based Differential Proteomics Technology. Tat protein was identified by matching the calibrated peptide mass values within NCBInr protein databases and a Tat peptide sequence ***LEPWKHPGSQPK*** was identified by the MS/MS spectrum, confirming the presence of Tat protein in extracts prepared from uninfected primary human neurons treated with supernatant from HIV-infected cells. To validate our data, we performed a Western blot analysis using anti-Tat antibody, however, we were unable to detect Tat protein due to its small amount (data not shown, ref. 10).

Since we have detected Tat in neuronal extracts, we next examined its functionality using transfection of the HIV-1 LTR reporter plasmid by nucleofection. Nucleofector technology allows gene expression to start immediately, independently of cell division. *Thus nucleofected cells can be analyzed after an extremely short incubation time*. Human microglia and neuronal cells were transfected with 0.5 µg of the HIV-1 reporter plasmid LTR-luc. 1 µg of CMV-β-gal was also included. After 24 h, rTat was added (1 µg, 1 ng, or 1 pg/ml) followed by luciferase assay. rTat up regulates the HIV-1 promoter in both cell lines (Fig. 2B, compare columns 2–4 to 1 and 6–8 to 5). The functionality of rTat was also determined in the neuronal cell line SH-SY5Y stably transfected and expressing LTR-luc-IRES-GFP, which contains 2 reporter genes, luciferase and green fluorescent protein (GFP) separated by an internal ribosome entry site (IRES). SH-SY5Y cells were transfected with 0.5 µg of Tat expression plasmid (Fig. 2C, lane 2), or infected with 1 MOI of Ad-null (lane 3) or Ad-Tat (lane 4). Cells were also treated with 1 pg/ml of rTat (lane 5) or with supernatant from U937 cells infected with the JR-FL strain of HIV-1 (lane 6). Luciferase assay shows that Tat activates the HIV-1 promoter in neuronal cells compared to mock (Fig. 2C, lane 1) or Ad-null (lane 3). Activation of LTR by Tat ranged from 2-fold with rTat (lane 5) to 13 fold with supernatant from infected cells (lane 6). These data demonstrate the functionality of rTat, i.e., 1 pg/ml of rTat protein activates the HIV-1 promoter. Our results correlate with the previous reports of Tat activation of the HIV-1 LTR in neuronal cells [22].

**Figure 3:**
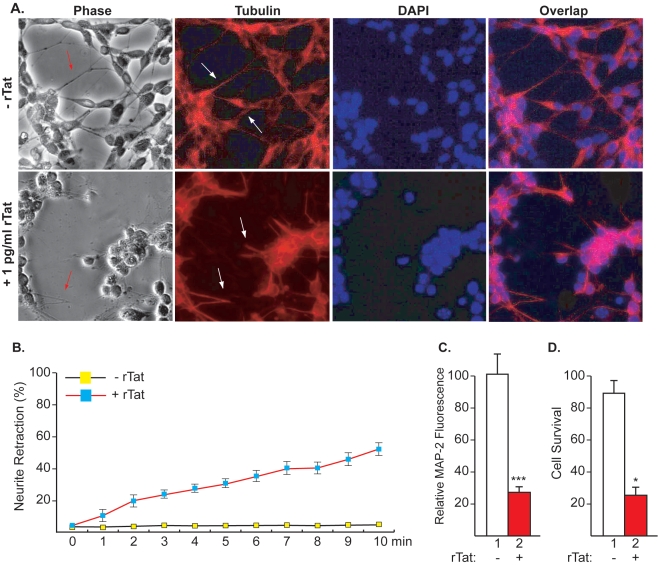
rTat cause dendritic retraction in SH-SY5Y. ***A***. Phase contrast microscopy of SH-SY5Y cells either untreated or treated with rTat as indicated. The cells were stained with DAPI (blue) and anti-tubulin (red) to mark the nucleus and the processes, respectively. ***B***. Tat-induced neurites retraction is time-dependent. Cell cultures were exposed to 1 pg/ml of rTat and assessed over 10 min. Values are expressed as percentage neurites retraction. *P*<0.01 vs control (untreated) (ANOVA, followed by Bonferroni test). Values represent mean (SD), (n = 15). ***C***. Quantification of MAP-2 immunoreactivity revealed marked reduction (75%) in rTat-treated SH-SY5Y cells. ***D***. Effect of rTat-treatment on SH-SY5Y neuronal death as assessed by trypan blue exclusion.

Next, we examined whether Tat affects neurites retraction, we treated SH-SY5Y cells with rTat (1 pg/ml) for 24 h in duplicate where one set was used to determine neurites retraction while the other set was used to determine impact of Tat on microtubules. Using anti-tubulin and DAPI staining to detect the neurites and the cellular nuclei, respectively, we observe that treatment of the neurons with Tat resulted in neurites retraction relative to untreated cells (Fig. 3A). These findings agree with previous reports where 2 - 20 femtomoles of Tat protein were shown to be neurotoxic [8], [23]. Importantly, our studies show a higher concentration of Tat in neurons derived from the supernatant of infected cells.

To study the effects of Tat on SH-SY5Y neurites, we used time-lapse microscopy. The response dynamics were evaluated by measuring neurites retraction distance. This was compared with the initial length of the extended neurite, which varied between 4 and 25 µm. Images of retracted neurites show that most of them are characterized by a retraction bulb and a thin trailing remnant (panel A, arrows). To determine the time course of neurites retraction, we measured neurite length over 10 min after rTat application. Between 1 and 2 min after exposure to 1 pg/ml of rTat, neurites had retracted by 10.3% and 19.7%, respectively [mean (SD)], *p*<0.01 compared with the untreated neurites that showed almost no change in length. Neurites continued to retract over time such that at 10 min post-Tat application neurites retraction was almost 52.1%.

Alternatively, we sought to further examine the impact of Tat treatment on microtubules using the second set of cells treated with 1 pg/ml of rTat protein led to a marked reduction (75%) in the Microtubule-associated protein 2 (MAP-2) reactivity (Fig. 3B), compared with untreated cells (compare lanes 1 and 2). Note that MAP-2 is a protein that belongs to the microtubule-associated protein family and serve to stabilize microtubules (MT) growth [24]. These results complement and confirm the data observed in panel A with anti-tubulin regarding alteration of microtubules.

Finally, we investigated whether addition of Tat leads to cell death. To that end, SH-SY5Y cells were treated with 1 pg/ml of rTat for 24 hrs after which the cells were stained with trypan blue and cell death was assessed. Briefly, five cell areas were used (each contains ∼100 cells) to count the number of dead cells. Interestingly, there was approximately 4-fold increase in cell death as measured by trypan blue exclusion in Tat-treated neurons when compared to untreated cells (Fig. 3C, compare lanes 1 and 2) [25]. Results were significant using statistical analysis as described in the Methods section.

In summary, neuronal degeneration remains a major problem associated with HIV-1 infection of the CNS even with successful HAART. In this regard, several factors, including the viral protein Tat, are involved in the development of neuronal injury, however the exact amount of circulating Tat protein remains unclear. Despites the several attempts that aimed to measure the amount of circulating Tat in cells, in human sera as well as in CSF [8], [26], [27], the levels of circulating Tat protein remains unclear. Here using capillary electrophoresis, we provided evidence to show for the first time the presence and amount of viral protein Tat in neurons. Taken together, our data showed that released Tat protein can be taken up by neuronal cells and causes neuronal deregulation through a pathway that remains to be identified. In this regard, we recently demonstrated that Tat causes neuronal deregulation by increasing the levels of secreted calcium, mitochondria deregulation and axonal transport alteration (manuscript in preparation). Further, our data presented in Figure 3 showed that Tat affects the levels and eventually the function of MAP2, which in turn could lead to alteration of axonal transport and communication (Fig. 3A and B). Therefore, the new method developed in this study clearly demonstrated the limitation of the previous techniques used to measure Tat in neuronal cells. It will also help in determining the amount of other viral proteins in infected cells. Further identification and measurement of Tat in non-infected cells may also help in estimating the amount of antibodies that should be used to neutralize the effect of Tat, which could lead to the development of more efficient therapy.
